# 3-D generated anatomic custom talar cement spacers: case reports, technical tips and literature review

**DOI:** 10.1186/s41205-021-00117-5

**Published:** 2021-09-17

**Authors:** Kimberly K. Broughton, Bonnie Chien, Derek Stenquist, Caroline Williams, Christopher P. Miller, John Y. Kwon

**Affiliations:** 1 Department of Orthopaedic Surgery, Brigham and Women’s Hospital Brigham and Women’s Hospital, 1153 Centre St, 5 South, MA 02130 Boston, USA; 2 Harvard Combined Orthopaedic Residency Program Massachusetts General Hospital, 55 Fruit Street, MA 02114 Boston, USA; 3 Dept of Orthopaedic Surgery, Beth Israel Deaconess Medical Center Beth Israel Deaconess Medical Center, 330 Brookline Ave, MA 02215 Boston, USA

**Keywords:** Limb salvage, 3D Talus, antibiotic cement, talus arthroplasty, infected nonunion

## Abstract

**Background:**

With today’s expanding use of total ankle arthroplasty, the ever-present trauma patient, and patients with uncontrolled comorbid conditions, surgeons face significant challenges for lower extremity reconstruction. These patients highlight some of those who may present with unique anatomy, bone loss, infection, and various other local and systemic factors that affect treatment options for successful outcomes. Three dimensional (3-D) printing for medical devices is allowing for new and customized ways to meet patient and surgeon goals of limb salvage and reconstruction.

**Case presentations:**

While the majority of 3-D printing is done for the purpose of implantation, we present a technical tip for designing a 3-D printed mold from which to create an antibiotic cement spacer for implantation. With two case illustrations including a talus fracture nonunion and infected subtalar arthrodesis nonunion, we describe the process of patient selection, implant design, fabrication, and implantation of a custom molded antibiotic cement talus.

**Discussion:**

Case illustrations present two successful limb salvage patients while giving a thorough explanation of our technique, learned tips and tricks. This applied technology builds on prior use of antibiotic cement in limb salvage of the lower extremity, most of which are joint sacrificing. 3-D printing the mold for an anatomic talus cement spacer results in a joint sparing limb salvage solution.

Innovative 3-D printing technology is merged with current, pertinent literature regarding antibiotic cement to offer surgeons expanded options for temporary or definitive reconstructive techniques in some of the most challenging patients.

## Background

Three dimensional (3-D) printing is an actively emerging field within the realm of medical devices. This technology expands therapeutic options and customization of implants to individual patients. Regarding orthopedic implant design, the ability to create products that replicate normal anatomy has been a significant advancement in the setting of difficult clinical scenarios. 3-D generated, custom implants increase the potential for joint and limb salvage with the goal of improving functional outcomes.

We present a technical tip for utilizing 3-D printing technology to create custom, anatomically-matched cement spacers for limb-salvage applications. We present two illustrative cases including a talus fracture nonunion with infected total talus implant and an infected subtalar fusion nonunion with severe talus collapse where 3-D technology was utilized for limb-salvage. We share our experiences and lessons learned, as well as a succinct review of the pertinent literature merging 3-D printing and the use of antibiotic cement as temporary and permanent reconstructive solutions.

## Case presentations

### Case 1

Patient M.C. is a 32 year old male smoker involved in a motor vehicle accident who sustained a closed, displaced talar neck fracture. This was treated with initial external fixation and staged open reduction internal fixation (ORIF) in 2018.

**Fig.1:**
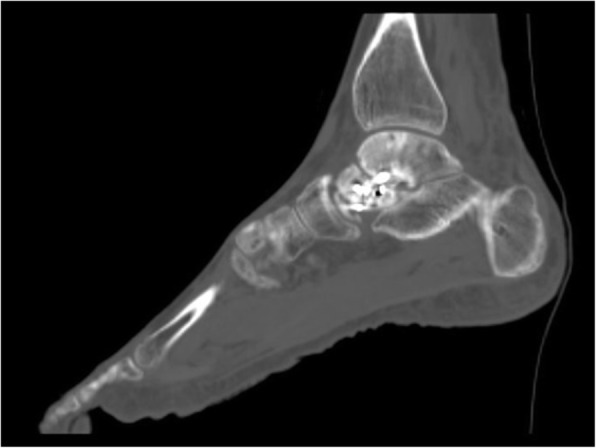
Preoperative sagittal CT demonstrating talar neck nonunion with associated post-traumatic ankle arthrosis and failed appropriate conservative modalities

He developed a nonunion with post-traumatic ankle arthrosis and failed appropriate conservative modalities (Fig. 1). Given his daily pain, disability and inability to continue employment as a construction worker he was indicated for surgical intervention. Multiple surgical options were considered. He had a history of heavy tobacco use but was willing to abstain preoperatively. However given this history, attempted bone grafting and ORIF or hindfoot arthrodesis in any form were considered to be at significant risk for nonunion. Given the maintained adjacent joint surfaces, a custom total talus replacement was felt to be the best clinical option in this highly comorbid patient.

**Fig.2:**
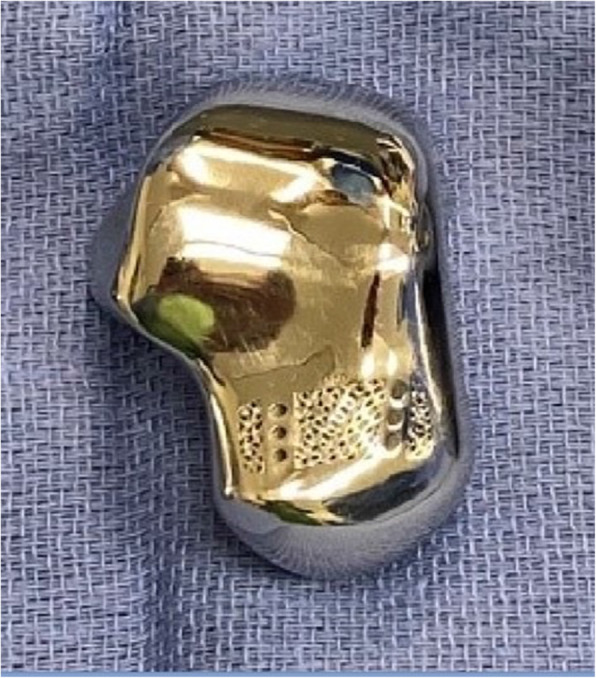
Custom 3D printed anatomically-matched custom cobalt chrome right talus dorsal view (restor3D Inc. Durham, NC)

Bilateral ankle computed tomography (CT) scans were performed preoperatively. An anatomically-matched custom cobalt chrome right talus was designed and manufactured (restor3D Inc. Durham, NC) based off 3-D reconstructions from the contralateral normal talus (Fig. 2). Three sizes were provided including an exact size match, and 95 and 105 % volume size renderings. A channel was designed in the dorsal neck to accommodate the curve of a circle taper-2 needle for soft tissue and capsular closure.

**Fig.3:**
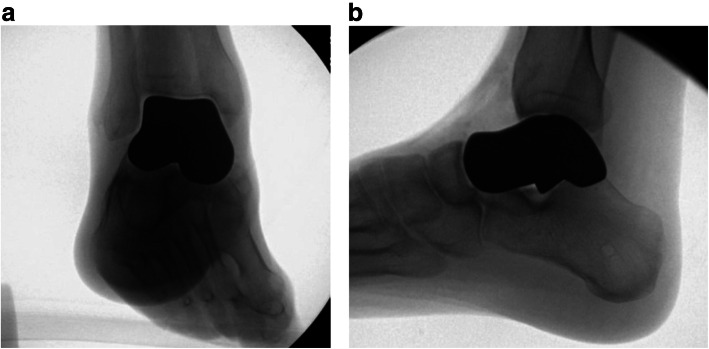
a) Mortise and b) Lateral intraoperative images after total talus replacement

After a period of preoperative smoking cessation and optimization, he underwent successful right talectomy and total talus replacement (Fig. 3). He did well in the initial postoperative period but presented with a small wound dehiscence 6 weeks post-surgery. Conservative wound care was trialed but failed and there was increasing drainage, dehiscence and concerns for deep infection. He underwent irrigation and debridement with the prosthesis retained. Deep cultures revealed a methicillin-sensitive Staphylococcus aureus and Streptococcus anginosus infection and he was placed on appropriate intravenous (IV) antibiotic therapy per the consulting infectious disease service. Negative pressure wound therapy was applied and continued upon hospital discharge.

**Fig.4:**
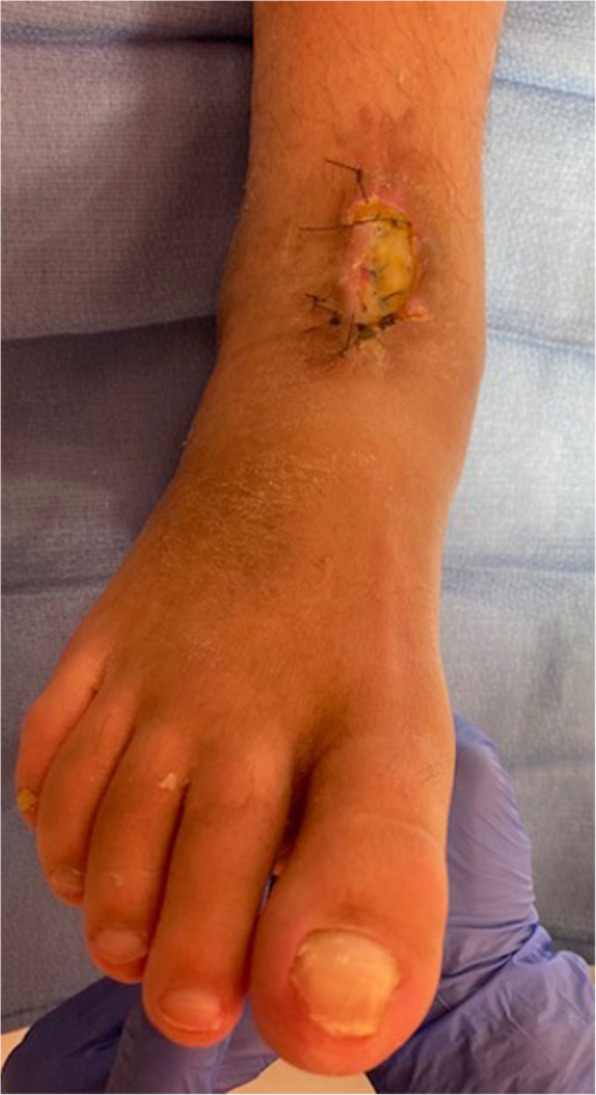
Continued wound dehiscence probing to the prosthesis with exposed tendon. Operative deep cultures revealed a methicillin-sensitive Staphylococcus aureus and Streptococcus anginosus infection

**Fig.5:**
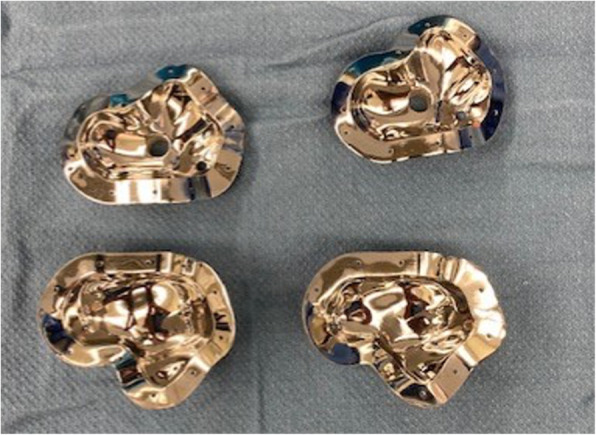
3D printed molds in 95% and 100% size renderings displayed open. Cylindrical ports for cement injection and air vacuum line seen on the plantar half of the mold.

2 weeks postoperatively he displayed poor wound healing and recurrent dehiscence (Fig. 4). Multiple options were discussed with the patient and he wished for attempted limb salvage. Based on previous 3-D modeling, a custom cobalt chrome mold was produced (Restor3D Inc., Durham, NC) to allow molding of an antibiotic-eluting cement talus spacer matching his total talus prosthesis (Fig. 5).

**Fig.6:**
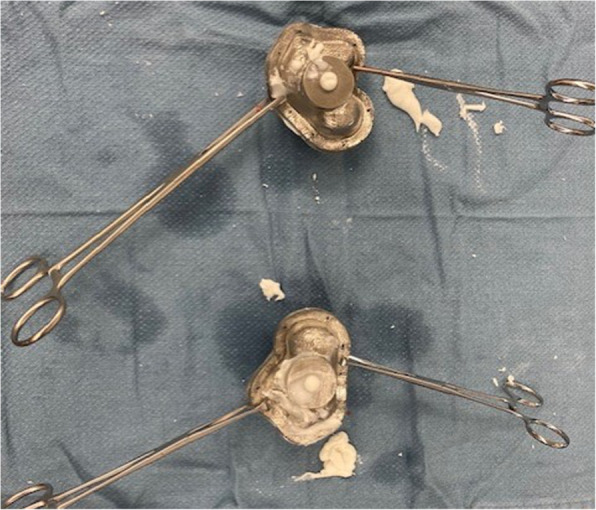
Kocher clamps holding the clamshell mold together while the cement cures. The antibiotic-eluting cement talus spacer was created intraoperatively using tobramycin-laden bone cement (Simplex, Stryker, Kalamazoo, MI) with 2 grams of vancomycin powder added

**Fig.7:**
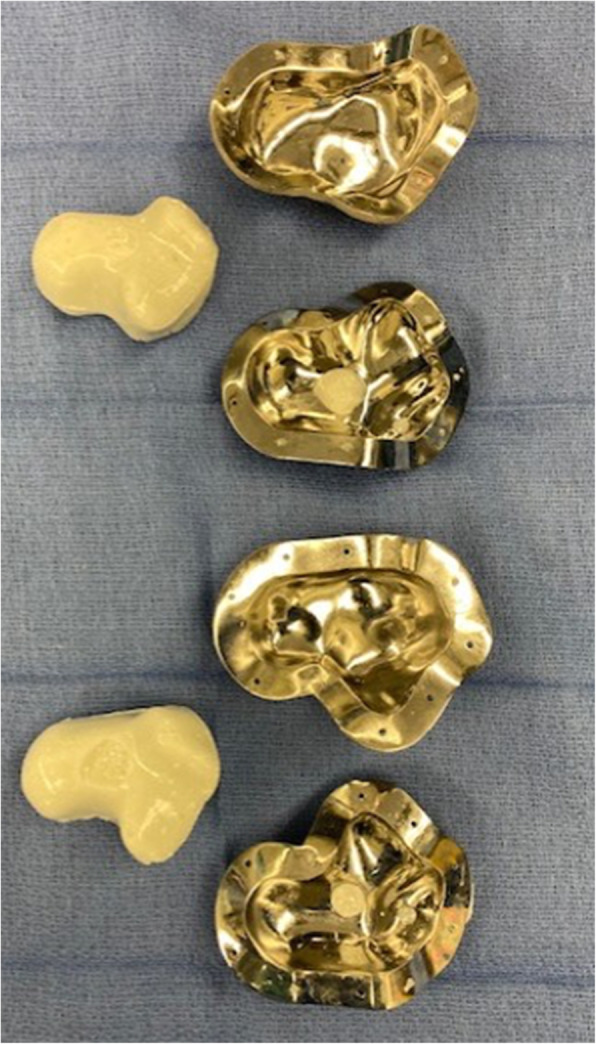
Molds open with resultant tobramycin-laden bone cement (Simplex, Stryker, Kalamazoo, MI) plus 2 grams of vancomycin cement spacer in 95% and 100% sizes

**Fig.8:**
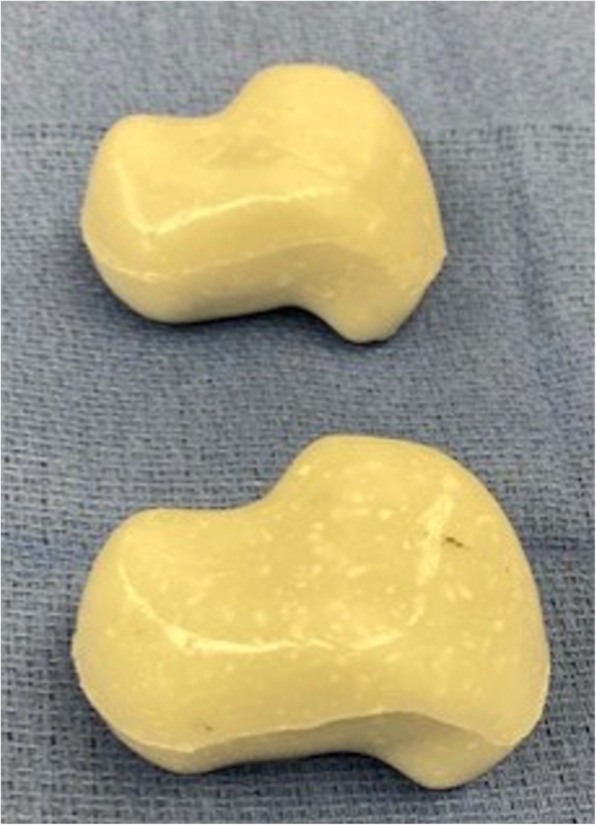
Final rendering of antibiotic cement spacers

**Fig.9:**
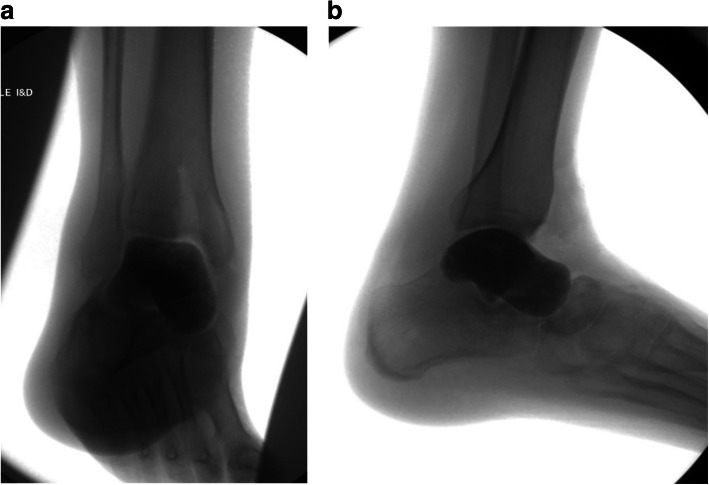
Mortise (a) and lateral (b) fluoroscopic images of 95% cement spacer in place

The patient returned to the operating theatre for explantation and repeat irrigation and debridement. The antibiotic-eluting cement talus spacer was created using tobramycin-laden bone cement (Simplex, Stryker, Kalamazoo, MI) with 2 g of vancomycin powder added (Figs. 6, 7 and 8). The 95 % volumetric sized spacer was utilized to facilitate insertion and prevent overstuffing of the cavity. There were no accessible sites for capsular or soft tissue attachment to the spacer built into the design, but the mortise and midfoot were noted to be stable with direct visual and fluoroscopic stress view evaluations (Fig. 9 A,B). IV antibiotic therapy was continued and plastic surgery was consulted. The patient underwent free flap coverage and went on to successful healing. The patient did not require any additional surgical procedures and was seen for final follow up approximately 11 months after his last procedure. He was weightbearing as tolerated in a regular shoe and despite reporting 3/10 daily pain was able to work in a floral warehouse and perform light cardiovascular exercises. While his preoperative range of motion (ROM) was not documented, he displayed approximately 10 degrees and 25 degrees of ankle dorsiflexion and plantarflexion respectively as measured by a goniometer. He demonstrated approximately 15 degrees of combined inversion/eversion subtalar motion.

### Case 2

**Fig.10:**
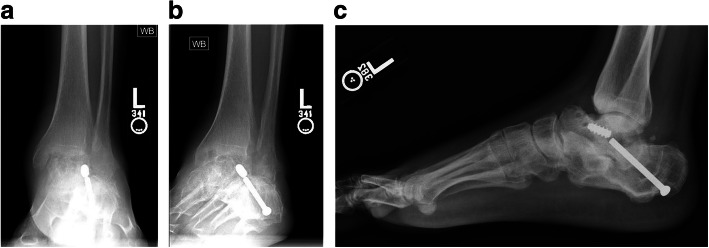
Case 2 preoperative AP (a), Mortise (b) and lateral (c) radiographs significant for a subtalar nonunion, broken hardware, and destruction of the talus consistent with an infected nonunion of the subtalar joint and osteomyelitis

Patient LC is a 57 year old male smoker with Hepatitis C, who underwent primary subtalar fusion in 2001 after a fall from a height. He had no known postoperative complications, but never had resolution of pain. He had a spontaneous eruption of purulent drainage from the wound 10 years post operatively which was treated with debridement, irrigation and IV antibiotics. His wound healed, but he continued to have exquisite pain limiting weight bearing. His presenting clinical examination to our institution was notable for erythema and warmth along the lateral ankle. Imaging was significant for a subtalar nonunion, broken hardware, and destruction of the talus consistent with an infected nonunion of the subtalar joint and osteomyelitis (Fig. 10 A-C). Given evidence of continued infection in this 57 year old smoker, his options for limb salvage were exceptionally limited. The options and associated risks were thoroughly discussed with the patient. The decided plan was for talectomy, extensive irrigation and debridement, and anatomic antibiotic cement talus spacer placement. A contralateral hindfoot CT scan was utilized to produce a custom cobalt chrome mold of the inverse contralateral talus (restor3D Inc. Durham, NC) which would restore height and alignment of his hindfoot while providing concentrated local delivery of antibiotics.

He was lost to follow up for 3 months at which time, he presented to the ED with bacteremia and elevated laboratory markers/indices. He was admitted to the hospital, started on IV antibiotics, and taken to the operating room the following day for talus excision, debridement, and 3D custom molded antibiotic cement spacer placement.

**Fig.11:**
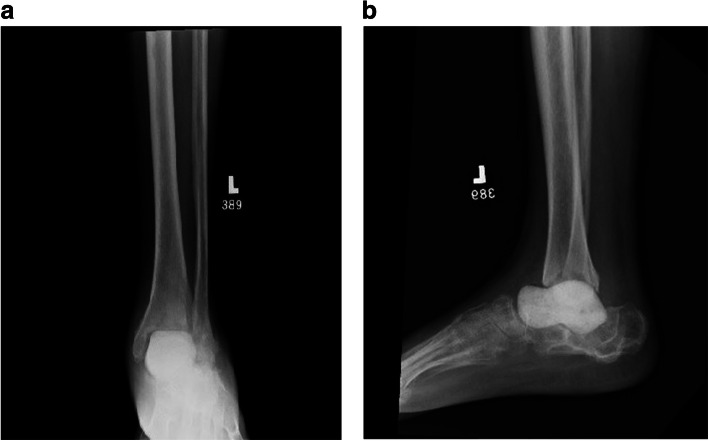
Case 2 postoperative AP (a) and lateral (b) radiographs with anatomically matched antibiotic cement spacer

Intraoperatively, he had marked erosion of the native talus which was removed along with the surrounding fibrotic tissue to the level of the calcaneus. Multiple tissue specimens were sent to microbiology and pathology. Care was taken to avoid injury to the tibia plafond chondral surface. The 95 % volumetric rendering sizer had excellent fit without overstuffing or allowing excessive hindfoot motion. Dorsiflexion was improved with a percutaneous tendoachilles lengthening. Tobramycin-laden bone cement (Simplex, Stryker, Kalamazoo, MI) with 2 g of vancomycin powder was vacuum mixed and injected into the mineral oil-coated molds. After hardening, the cement spacer was removed from the mold, edges smoothed, and implanted into the patient (Fig. 11 A, B). The wound was closed in layers and an incisional wound vac was applied for 48 h post operatively. He was initially immobilized and nonweightbearing to allow for soft tissue healing, but at four weeks post operatively, he began progressive motion and weightbearing. The patient went on to successful healing with eventual eradication of polymicrobial infection after a prolonged course of IV antibiotic therapy. The patient did not require any additional surgical procedures and was seen for final follow up approximately 4 months after his last procedure. He was weightbearing as tolerated in a regular shoe and reported 3/10 daily pain. His incisions were well healed without erythema nor drainage. He continued to be unemployed. While his preoperative ROM was not documented, he displayed approximately 6 degrees and 20 degrees of ankle dorsiflexion and plantarflexion respectively. He demonstrated approximately 10 degrees of combined inversion/eversion subtalar motion. While repeat clinical examination was recommended, he was thereafter lost to follow-up.

### TECHNIQUE: Production of the 3-D Generated Anatomic Custom Talar Cement Spacer Mold

CT scanning was performed in accordance with computer aided design (CAD) parameters. Notably, slice spacing less than 1.25mm (but up to 1.5mm) is recommended with pixel size of 0.5mm or less. The field of view should always include all relevant anatomy plus limited additional extension to allow for guide wrapping or implant fixation per preoperative planning. The studies were in DICOM files and within a timeframe where no significant change in patient anatomy had occurred.

3-D rendering of patient anatomy often requires imaging the contralateral side. In these chronic and destructive processes, the contralateral limb CT scan can be inverted by computer design software to provide an anatomic matched rendering. We used patient 1’s contralateral side for original 3-D talus implant. This design was further utilized to render his 3-D printed cement mold. Patient 2’s inverted, contralateral talus was also used to create the mold representing the negative impression. Patient 2’s prior infected nonunion of attempted subtalar arthrodesis created a nonanatomic calcaneus articulation; however, to restore height and alignment, recreating a fully anatomic talus from the contralateral side was considered most appropriate and more predictable than attempting to know the exact extent of required surgical debridement.

The molds were fabricated by selective laser melting (SLM) of cobalt chrome alloy (CoCrMo). CoCrMo was chosen since it can be highly polished. This highly polished internal surface was important to minimize irregularities in the antibiotic spacer surface as well as facilitate release of the spacer from the mold. The SLM additive manufacturing process uses raw material in powder form and builds up three-dimensional parts with successive two-dimensional layers based on a CAD model. For each layer, the powder is spread across a build plate in a 20–100 μm thick layer, then a high energy laser selectively melts the powder as defined by the CAD, fusing together the material. This process is repeated layer by layer to manufacture the final part.

The mold for Case 1 was designed with two cylindrical ports. One for injecting the cement, sized to match the outer diameter of the syringe used for insertion. The second provided a vacuum line to remove air from the cavity to minimize potential air pockets within the cement. A non-planar parting line was designed to enable the removal of the spacer despite its complex surface geometry. Case 2’s mold utilized the similar non-planar parting line; however, only one portal was used. The portal was large enough to allow for both syringe insertion of cement and air escape. It was placed along the partition line of the two halves of the mold with intention of facilitating cement spacer removal from the mold. Both cases had multiple CoCrMo molds printed including size-matched, 105 and 95 % volume versions. Sterile mineral oil was used to lubricate the internal surfaces to aid in release (Figs. 5, 6, 7 and 8).

### Surgical/technical considerations

There are technical issues to consider during preoperative planning and intraoperative execution. Removal of the custom total talus metal prosthesis was difficult due to the implant matching normal anatomic shape/size and without means of piecemeal removal. It is the authors’ recommendation to have complete muscle paralysis induced by the anesthesia team which allows for increased ankle distraction. A femoral distractor or large external fixator should be available for additional opening of the periprosthetic space. If a custom implant is being used, the possibility of future removal should be considered during its original fabrication. Given the possibilities allowed by 3-D printing, threading to allow for insertion of an extraction device should be considered. Other company-specific implants may have proprietary extraction tools, and these should be available at the time of surgery.

For production of the cement spacer, the cement should contain heat-stable antibiotics that address all known pathogens. Potential patient allergies to antibiotics should be verified to ensure no adverse reactions. Preoperative consultation with the infectious disease service is recommended. Vancomycin is heat stable and covers most strains of common gram positive (such as Staphylococcus and Streptococcus species) pathogens. Cement pre-loaded with antibiotic typically uses an aminoglycoside such as tobramycin or gentamycin which serve to broaden gram negative antimicrobial coverage.[1] Further antibiotic considerations can be extrapolated from recommendations by the 2018 International Consensus Meeting of Musculoskeletal Infection specific to foot and ankle.[1] In our cases, each mold required 2 packages of cement to which we added 2 g vancomycin. The cement powder and vancomycin were mixed thoroughly before adding the liquid monomer. The cement was mixed in a vacuum hand mixer to minimize air pockets or other impurities. While still viscous, the cement was poured into a 60 cc syringe and then injected into the inlet port of the mold until cement overflowed all ports indicating that the mold had been maximally filled. The mold was clamped externally to keep it tightly closed and ensure pressurization of the cement and maintaining the spatial resolution accuracy of 100 μm as per the mold’s design and printing. The cement talus was removed from the mold once the sample cement had hardened.

Port placement on the mold deserves particular consideration. We present two different port configurations giving attention to sizing adequate to accommodate a 60 cc syringe nozzle end and that has room for air to escape. The ports should be placed on non-articulating areas of the cement spacer and for two port design, we recommend they be parallel to facilitate removal of the cement spacer. Other instruments on hand to ‘trim’ the cement talus should include rongeurs, osteotomes, rasps and a microsaggital saw in the event of significant residual cement pillars extending from the port sites.

Lastly, planning of surgical personnel is important in a multi-step procedure such as this. Consider dividing the surgical team into two to allow for simultaneous work: one team can perform the surgical approach, implant/bone removal and irrigation and debridement while the other works on the back table to produce the custom cement spacer.

## Discussion

We present two illustrative cases demonstrating utilization of 3-D generated prostheses for limb salvage in the treatment of end-stage talar pathology complicated by infection. While the use of 3-D printing to create custom implants is emerging, little has been written regarding its use to create custom antibiotic cement spacers to enhance reconstructive and salvage solutions for the foot and ankle.

Antibiotic-laden cement is widely used across orthopaedics. Most often, it is used as a bridge in an infected revision/reconstructive procedure acting as a physical space holder and as an antibiotic carrier directly to the affected tissues. Literature discussing cement spacers for permanent implantation in the foot and ankle is limited to case reports and short case series. Hong et al. reported a successful outcome of modified hindfoot fusion and midfoot antibiotic cement spacer with overlying adipofascial flap coverage in a 53 year old woman with severe Charcot destruction and ulceration. [2] Forefoot osteomyelitis treated similarly had encouraging outcomes as reported by Melamed et al. in their series of 23 patients. [3] The cement spacer was molded in situ to fill the defect and was left permanently in nearly 50 % of patients. The locations of retained spacers included the hallux interphalangeal joint, the 1st metatarsal head, and the metatarsophalangeal joint. The overall success rate was 91.3 % at an average of 21 months, defined by the authors as resolution of infection without the need for amputation. Elmarsafi and colleagues reached similar conclusions regarding the use of cement spacers for limb salvage in diabetic patients with osteomyelitis of the mid and forefoot. They documented a 66.7 % salvage rate in 30 high-risk diabetic patients with an average follow up of 52 months and concluded that cement spacers are safe and durable and provide a viable definitive reconstructive option. [4]

Nonanatomic cement spacers for the tibiotalar joint have been reported as definitive treatment with mixed results. Ferrao et al. described their experience treating postoperative ankle infections after total ankle replacement (TAR) or arthrodesis.[5] Their series included 6 patients with infection after TAR and 3 patients after arthrodesis that were either medically unfit for further surgery or preferred to retain their spacer. Two patients required below knee amputation for recurrent infection while the remaining seven patients with retained cement spacers were able to perform basic activities of daily living with minimal discomfort at an average of 20.1 months (range 6–62 months).[5] They noted a tendency for spacer loosening and migration, although no patient demonstrated excessive bone loss at final follow up. The spacers were shaped by hand in situ, and the authors emphasized the importance of limiting cement protrusion anterior to the tibia to avoid pressure on the skin.[5] Lee and colleagues reported on functional outcomes after cement arthroplasty as the primary salvage procedure for ankle joint destruction.[6] The authors followed 16 patients with intractable infection, nonunion, and/or a large bone defect or tumor requiring a cement spacer for an average of 39 months (range 14–100). Two patients demonstrated subluxation and osteolysis at 10 and 78 months. Mean AOFAS and VAS scores improved significantly from 39 pre-operatively to 70 post-operatively and from 8 to 3, respectively. Nine of 16 patients were able to walk continuously for more than an hour. The authors molded the cement by hand in situ, adding screws in some cases to augment stability and maintaining overall mechanical alignment of the joint manually while the cement cured. They concluded that primary cement arthroplasty is a viable option for elderly or low-demand patients.[6].

Custom antibiotic cement spacers have long been utilized in the treatment of prosthetic joint infection (PJI) in total hip and knee replacement.[7–10] While a complete review is beyond the scope of this work, more advanced spacer designs used in hip and knee surgery may demonstrate similar benefit for the foot and ankle. Articulating spacers have been associated with improved outcomes compared to static spacers in revision total knee replacement (TKR) for infection, possibly by preserving physiologic motion of the soft tissue envelope prior to reimplantation.[11] Patients with articulating spacers for PJI after total hip replacement (THR) had better functional scores than patients awaiting primary THR but lower than patients after successful primary THR in one Canadian study.[12] Several studies have described the use of antibiotic spacers as long-term or definitive treatment in the THR, TKR, and total shoulder replacement (TSR) literature.[11–14] Currently, there are no prefabricated articulating TAR spacers on the market, but the body of hip and knee literature on this topic is invaluable as revision TAR technology advances.

There are few investigations in the foot and ankle literature reporting increased customization of cement spacers for staged reconstruction of the tibiotalar joint after infection or trauma.[15, 16] Short et al. described their technique for intra-operative fabrication and placement of an articulating antibiotic cement spacer through an anterior approach in a patient with an infected TAR. The implant provided 10 degrees of dorsiflexion and 15 degrees of plantarflexion. Extrapolating from the arthroplasty literature, the authors suggested that articulating spacers for the foot and ankle might have similar advantages over static spacers such as delivering high doses of antibiotics locally while minimizing soft tissue contractures and maintaining some joint range of motion.[16, 17] In another case report, Huang and colleagues contoured a cement spacer by hand in a case of traumatic complete talar extrusion.[15] They used the available extruded talus to guide their back table fabrication of a replica antibiotic cement talus. This closely-matched replica talus was implanted and an external fixator applied in order to preserve leg length, joint space, and hindfoot alignment.^17^ Three months later, the patient underwent successful staged tibiocalcaneal fusion with femoral head interposition allograft.

Only one case report has documented the use of an anatomically-contoured total talus cement spacer as definitive treatment after total talus extrusion. Chiu and colleagues initially treated a 30-year old man for traumatic loss of the talus with an external fixator and a hand-molded antibiotic spacer intended to fill the void left by the talus.[18] Due to persistent instability, wound drainage, and infection, they produced an anatomically-molded spacer in the shape of the patient’s native talus by using a CT scan of the contralateral talus to 3D print a negative mold for the affected talus. Stability improved immediately after insertion of the anatomic spacer, and the patient was allowed full weightbearing approximately 5 weeks after removal of the external fixator. Infection was eradicated, and at 14 months’ follow up the patient was able to walk for 15 min without pain and had a 15-degree arc of motion at the ankle.

There are additional pragmatic considerations of custom printing, mainly cost and time sensitivity. Production of custom 3-D implants and molds may incur additional cost as compared to more traditional treatment options. However, this must be weighed in relation to the potential financial and personal costs incurred by the patient and health system of repeated surgeries, complications and lifetime prosthetic fitting if limb salvage is unachievable. For the illustrative cases described in this report, the total cost for fabrication of the custom produced cement molds was approximately $8,000 US Dollars. This is comparable to the cost of prefabricated knee or hip spacer molding kits currently available. While most conditions can be temporized by other surgical procedures until a custom 3-D implant is produced, the luxury of time may not be available in all scenarios. The total time, from the initiation of the design phase through final production of our custom cement molds, was less than 5 days. This turnaround time was expedited by utilizing the previously submitted CT imaging and with fluid communication between the surgeon and the design team. Of note, both the total talus implant and the cement molds are cleared for use under the FDA custom device exemption pathway as there are no commercially available predicates for these device indications.

Finally, the use of 3D-printed antibiotic spacers should be considered only when no other viable, time-tested options for limb-salvage remain. Although no supportive literature currently exists, joint preservation in the long-term is unlikely given the coefficient of friction between the cement/articular cartilage interface. While it is unclear whether custom 3-D printed cement spacers will be viable long-term options, we present it as a feasible option for acute limb-salvage. Furthermore, eradication of infection while maintaining normal joint space congruity may allow for future limb-length preserving reconstructive options, whether joint sparing or sacrificing, in some patients where amputation may seemingly be the only available immediate treatment option.

Custom 3D-printed antibiotic spacers have the potential to become an important solution for limb salvage in-particular for the tibiotalar joint. While further investigations are required, anatomically matched talar cement spacers may allow for improved joint kinematics and decreased osteolysis while allowing for the local delivery of antibiotics and preservation of joint space and function. Furthermore, increased use as definitive treatment without the need for secondary reconstruction may be possible due to their anatomic design which may improve function and pain relief as compared to nonanatomic spacers. As utilization of TAR is increasing,[19] there will be an increased need for revision and salvage solutions due to implant loosening and infection. Complications of post-traumatic conditions of the hindfoot such as osteonecrosis and infection may require similar solutions. Utilization of currently available 3-D printing technology as applied to production of anatomic cement spacers should be considered to enhance limb salvage. The bony defects resulting from TAR failure or post-traumatic collapse can lead to unique anatomy where preserving bone is paramount for future revision and reconstruction.

3-D printing has the potential to be a useful tool for temporary or long term solutions in these scenarios. While joint preservation in the long-term is unlikely given the coefficient of friction between the cement/articular cartilage interface, this technique may allow for limb-salvage or temporization until comorbid conditions may be optimized to allow for other reconstructive procedures. Further studies are required to demonstrate cost-effectiveness in the setting of patient outcomes.

## Data Availability

Data sharing not applicable to this article as no datasets were generated or analyzed.

## Abbreviations

3D: Three dimensional
ORIF: open reduction internal fixation
CT: Computed tomography
IV: intravenous
SLM: Selective laser melting
CoCrMo: Cobalt chrome alloy
CAD: Computer aided design
TAR: total ankle replacement
AOFAS: American Orthopaedic Foot and Ankle Society
VAS: Visual analog scale
PJI: Prosthetic joint infection
TKR: Total knee replacement
THR: Total hip replacement
TSR: Total shoulder replacement
